# Modelling the Influence of Foot-and-Mouth Disease Vaccine Antigen Stability and Dose on the Bovine Immune Response

**DOI:** 10.1371/journal.pone.0030435

**Published:** 2012-02-17

**Authors:** David Schley, Reiko J. Tanaka, Kritsada Leungchavaphongse, Vahid Shahrezaei, John Ward, Clare Grant, Bryan Charleston, Christopher J. Rhodes

**Affiliations:** 1 Institute for Animal Health, Pirbright, Woking, United Kingdom; 2 Department of Bioengineering, Imperial College London, London, United Kingdom; 3 Department of Mathematics, Imperial College London, London, United Kingdom; 4 Department of Mathematical Sciences, Loughborough University, Leicestershire, United Kingdom; 5 Institute for Mathematical Sciences, Imperial College London, London, United Kingdom; Albert Einstein College of Medicine, United States of America

## Abstract

Foot and mouth disease virus causes a livestock disease of significant global socio-economic importance. Advances in its control and eradication depend critically on improvements in vaccine efficacy, which can be best achieved by better understanding the complex within-host immunodynamic response to inoculation. We present a detailed and empirically parametrised dynamical mathematical model of the hypothesised immune response in cattle, and explore its behaviour with reference to a variety of experimental observations relating to foot and mouth immunology. The model system is able to qualitatively account for the observed responses during in-vivo experiments, and we use it to gain insight into the incompletely understood effect of single and repeat inoculations of differing dosage using vaccine formulations of different structural stability.

## Introduction

Foot and mouth disease virus (FMDV) causes a highly infectious disease of cloven-hoofed animals that has significant global socio-economic impact. Foot and mouth disease (FMD) severely affects the welfare and productivity of high-value farm animals that are important to food security, including cattle, sheep and pigs ([1]. Disease-free status allows countries to participate in free trade of animals and produce. However, the loss of such status, even temporarily, can result in significant economic losses [2]. In countries where FMD is endemic the disease results in enormous losses, especially to small-scale livestock farmers, and it is ranked in the top ten livestock diseases for cattle and pigs in terms of impact on the poor globally [3].

Vaccination is one of the principle methods available for disease control and eradication, and mathematical modelling has been identified as playing a vital role in helping formulate effective strategies [4]. Whilst there has been much epidemiological modelling of FMD outbreaks and associated intervention strategies (see for example reviews [5], [6], [7]) this has been on a regional scale and hence at the farm-level. There exists little work focussing on the within-host dynamics of infection with wild-type virus, or modelling of immune responses to either infection or vaccination, although data from complex experiments able to elucidate this are now available [8], [9], [10].

FMD occurs as seven main serotypes (O, A, C, SAT1, SAT2, SAT3, Asia1) with numerous antigen subtypes within each strain. Vaccines tend to be most effective against the specific strain they are designed to elicit protection. However, there is an urgent need to develop better FMD vaccines which protect against a wider range of strains and, more importantly, confer longer-lasting host protection than existing formulations. Commercially available FMD vaccines are based on inactivated virus grown in large-scale cell culture. In many commercial livestock herds repeat vaccination is necessary to sustain host protection and, although costly, this approach is used in many parts of the world where FMD is endemic or sporadic. Consequently, improved vaccines would contribute significantly to reducing the economic burden imposed by FMD and improving food security.

Generating more effective FMD vaccines depends critically on developing a better understanding of the basic host immunological responses both to infection by wild-type virus strains and to vaccination with antigenic formulations. Much detailed experimental work on immune mechanisms has been undertaken *in-vivo* and *in-vitro* and has generated a wealth of insight into various aspects of host innate and adaptive responses (see for example references in [11].

However, key issues relating to vaccine immunogenicity remain unresolved. Juleff *et al.* [12] suggest that the repetitive antigenic sites embedded in the stable conformational structure of the viral capsid (by virtue of the icosahedral symmetry) are essential to stimulate a protective immune response to FMDV. Soluble antigens, disrupted capsids or peptides are incapable of inducing an equivalent response. The FMD virus is notoriously unstable, a likely consequence of infectivity relying on acid-induced capsid disassembly in endosomal vesicles following virus uptake from the cell surface; although there is some variation in stability across the serotypes [13]. As a result FMD vaccines are unstable in various environmental conditions, including mild acidic pH and elevated temperatures (as when the cold chain is broken). The instability of the icosahedral capsid is manifest as dissociation into smaller pentameric assemblies with a consequent loss of immunogenicity and this is an important concern for all FMD vaccines. Increased FMD vaccine stability is therefore a highly sought goal, not only for enhanced storage characteristics but also to increase duration of immunity. Using a mathematical model we are able to investigate the effect that vaccine stability has on within-host adaptive immune responses. Here adjuvant is included implicitly in the model, since the vaccine doses we are investigating are based on the observed properties of commercial veterinary preparations. The benefits these bring are generally assumed to have been optimised, but what is of interest here is it working within the known limitations of existing products. Moreover, using the model it is possible to explore the interplay of varying dose, repeat vaccination frequency and vaccine stability, all of which are features of vaccination protocols in veterinary practice. In doing this we exploit previous experimental work on FMD immunology, and moreover, formalise proposed immune response mechanisms against FMD in a mathematically consistent framework.

Specifically, we develop and parametrise a detailed dynamical model of the proposed within-host adaptive immunological response mechanism to inoculation with vaccine formulations of differing structural stability. The model is able to qualitatively account for empirically observed dynamics of the various constituent cell types in the coordinated immune response to the presence of antigen, as well as the generation of immune memory, thereby giving confidence that the proposed mechanism is an appropriate one. Moreover, it shows how repeat host vaccination and compensation of structural stability for dose can be used to maintain elevated levels of host protection. Results also indicate that capsid stability has no impact on the timing of the immune response though it has significant impact on its magnitude. Recent experimental work on antigen formulations that are either T-cell dependent or T-cell independent show a significant difference in the resultant immunoglobulin levels in cattle, and we show how to use the model to account for these observed immune dynamics. The specific effects of the stability and dose, individually and in combination, could not be clarified without developing a dynamical model that captures the essential components of the immune response.

The mathematical model complements existing experimental approaches to FMD immunology, and is intended to be used as a framework within which to formalise thinking about hypothesised immune mechanisms, and in the development of future experiments. Given the current level of knowledge with regard to bovine immunology, and the difficulties of deriving quantitative data on key factors from experiments, we believe that at present a coarse-scale model is the most appropriate for investigating this system.The model as it is presented and parameterised gives confidence that currently proposed immune mechanisms are sound, and furthermore it can be used as a point of departure to explore possible outcomes before additional experimental work is undertaken. In this way we aim to advance understanding of which potential future improvements in vaccine technology will be most efficacious.

**Figure 1:**
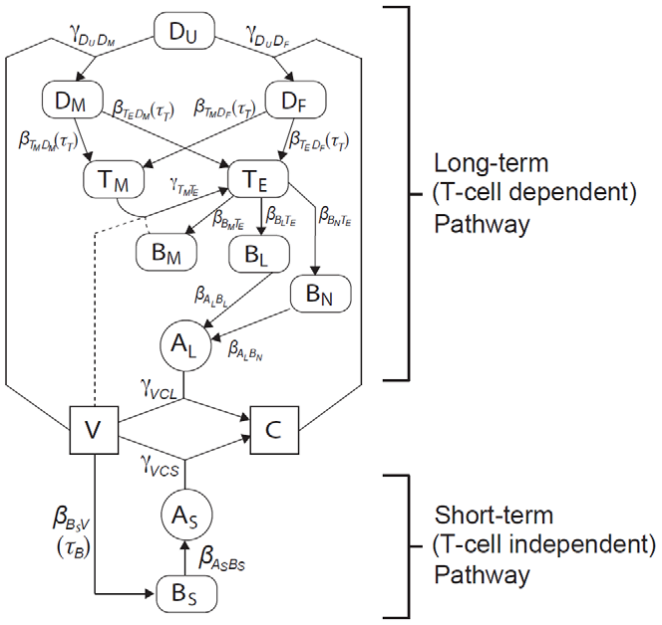
Schematic of the short-term and long-term dynamics of the immune response, as stimulated by vaccine antigen. For variable definitions see Table 1; for parameter definitions and values see Table 2.

We describe a hypothesised immune response to the presence of vaccine antigen (see Figure 1) and discuss viral capsid stability. The structure of the full immunological model and a review of the assumptions inherent in its development are presented in the Materials and Methods. Parameter estimates for the model are also presented here. The model is then used to explore the dynamics of the immune response to host inoculation with vaccine formulations of varying dose and stability, and these are discussed in relation to a variety of empirical and experimental observations. A model sensitivity analysis is also presented.

**Table 1:** Variables.

Variable	Interpretation
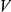	vaccine concentration
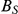	short term antibody secreting cell concentration
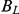	short term T-cell dependent antibody secreting cell concentration
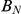	long term T-cell dependent antibody secreting (long-lived plasma) cell concentration
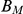	memory B cell concentration
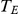	effector T cell concentration
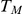	memory T cell concentration
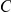	antigen-antibody complex concentration
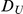	non-activated dendritic cell concentration
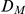	activated DC level via macropinocytosis
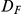	activated DC level via the FC activation process
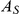	short term memory antibody IgM concentration
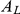	long term memory antibody IgG concentration

Definitions of system variables.

**Table 2:** Parameters.

Parameter	Interpretation	Value†	Source/justification
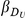	Dendritic cell migration rate	3.0 wk 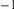	95% repopulation within 1 week after local removal
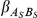	IgM production by short-term ASC rate	2.0 wk 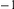	observed initial growth rate (see Figure 6)
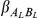	IgG production rate by short-term T-cell dependent ASC	”	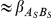
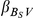	short-term ASC production rate	6.9 wk 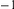	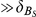
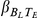	short term T-cell dependent ASC production rate	”	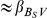
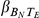	long-term T-cell dependent ASC production rate	”	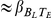
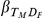	memory T-cell production by FC activated DCs rate	0.17 wk 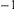	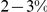 increase per day ([28]: p2 Table 1 – CD4 CD45R0)
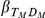	memory T-cell production by macropinocytosis activated DCs rate	”	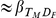
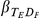	effector T-cell production by FC activated DCs rate	”	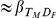
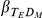	effector T-cell production by macropinocytosis activated DCs rate	”	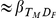
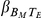	memory B-cell production rate	0.17 wk 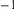	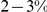 increase per day ([28]: p2 Table 1 – CD19 CD27+)
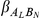	IgG production rate by long-term T-cell dependent ASC	1.36 wk 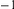	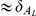
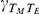	memory to effector T-cell conversion rate	1 wk 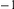 c 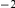	
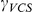	vaccine-IgM complex formation rate	2 wk 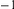 c 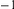	Observed in less than 1 hour
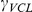	vaccine-IgG complex formation rate	”	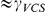
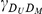	Vaccine uptake rate by micropinocytosis	1.4 wk 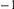	5% of final take up achieved within 6 hours (unpublished data)
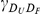	Complex uptake rate by FC activated pathway	19 wk 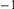	50% of final take up achieved within 6 hours (unpublished data)
ltirow2* 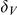 {	unstable vaccine decay rate	39 wk 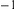	in-vitro half-life of 3 hrs [13]
	stable vaccine decay rate	19 wk 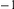	in-vitro half-life of 6 hrs [13]
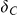	antigen-antibody complex decay rate	17 wk 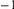	loss: 50% in 5 hrs, 90% in 30 hrs [29]
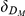	macropinacytosis-activated DC decay rate	13 wk 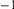	lifespan of 2–3 days (unpublished data)
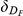	FC-activated DC decay rate	”	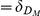
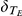	effector T-cell decay rate	4.7 wk 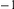	cleared within 1 week (unpublished data)
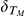	memory T-cell decay rate	0.51 wk 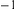	7% loss per day ([28]: p2 Table 1 – CD4 CD45R0)
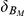	memory B-cell decay	0.43 wk 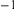	6% loss per day ([28]: p2 Table 1 – CD19 CD27+)
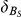	short-term ASC decay rate	0.69 wk 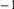	half life of 1 week (unpublished data)
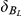	short-term T-cell dependent ASC decay rate	”	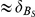
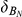	long-term T-cell dependent ASC decay rate	0.01 wk 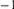	half-life of order one year
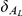	IgG decay rate	1.36 wk 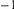	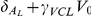 combined decay rate of 3.36 (see Figure 6)
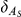	IgM decay rate	0.17 wk 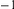	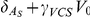 combined decay rate of 2.17 (see Figure 6)
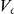	half-saturation of vaccine uptake by unactivated DCs	20 c	Saturation at 30–40 times standard dose (unpublished data)
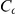	half-saturation of complex uptake by unactivated DCs	0.1 c	
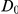	baseline non-activated dendritic cell concentration	1 c	
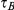	time-delay in B-cell response to vaccine	0.14 wks	observed within 1 day (unpublished data)
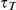	time-delay in T-cell response to activated DCs	0.57 wks	observed within 4 days (unpublished data)

Definitions of system parameters and values used in simulations (with justification and/or reference source). Here 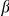 generally refers to production rates, 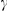 to conversion rates, 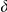 to decay rates and 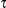 to temporal delays. For simulations we consider 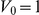.

† wk: week; c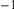: particle (vaccine, complex or cell) concentration.

## Results

The system of delay-differential equations (1)–(13), describing the dynamics of the variables listed in Table 1, was numerically integrated using the Runge-Kutta method, implemented through the dde23 routine in Matlab R2010a [14]. This permitted comparison of model outputs with experimental results. All simulations used the nominal parameter values given in Table 2, unless stated otherwise.

### Effect of vaccine dose and stability

**Figure 2:**
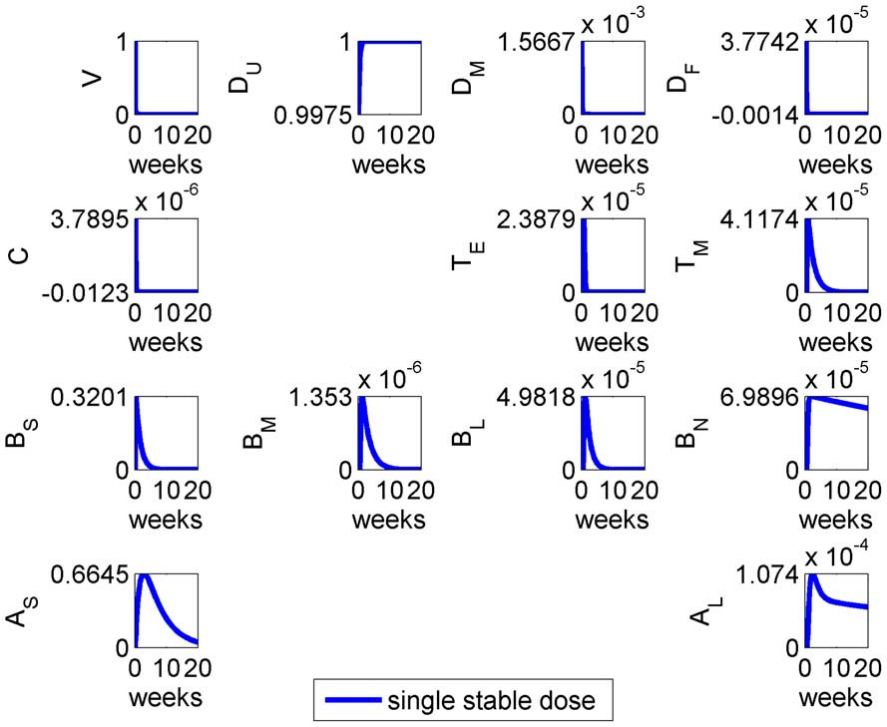
Model results for the system with initial vaccination at time 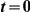, with parameters as given in Table 2 and “stable” vaccine.

The results of a single dose 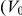) of stable vaccine antigen being introduced into the model system at time 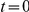 are given in Figure 2. The model simulation showed an immune response stimulated by vaccine antigen that lasted for several weeks: the timescales observed for the different cell types and antibodies were characteristic of those seen following immunisation of cattle [9].

Specifically the removal of vaccine antigen, 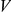, on a fast timescale was observed, together with the generation of a burst of short-term antibodies that peaked around the second week after immunisation. A longer-term antibody response 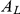 was generated, which persisted over the host lifetime, remaining present to immediately respond to future antigen challenges. A sustained, elevated, population of memory B cells and long-lived plasma B cells was also produced, which constituted the generation of immunological memory.

**Figure 3:**
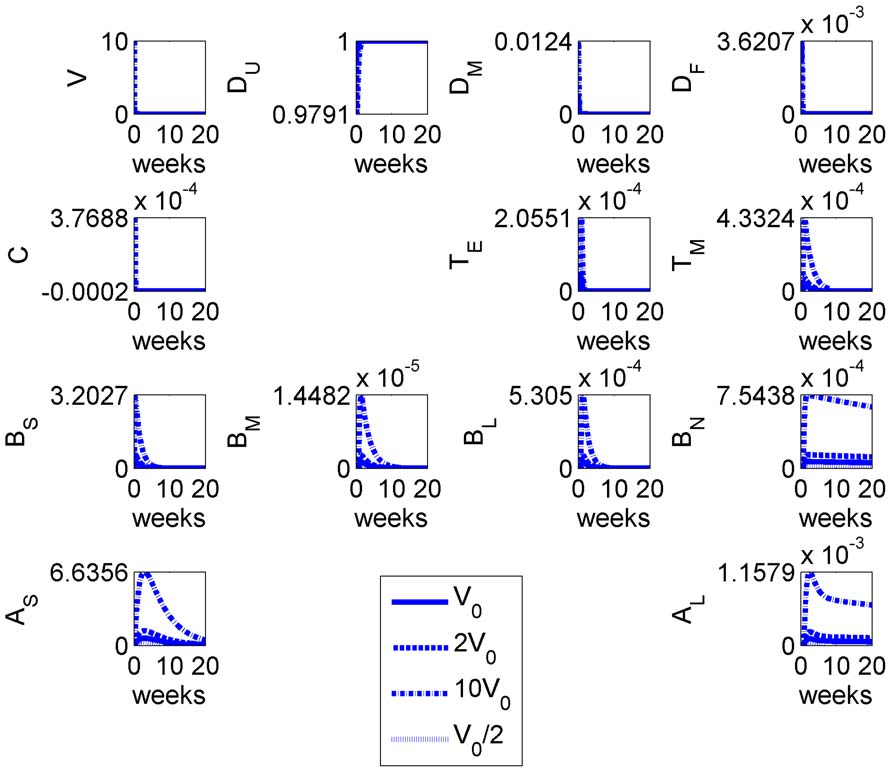
Model results for the system with different initial doses of (stable) vaccine: 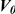 (solid), 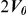 (dashed), 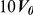 (dot-dashed) and 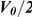 (dotted).

We repeated the calculation but varied the initial dose of (stable) vaccine antigen: results are given in Figure 3. The magnitude of the immune response was clearly sensitive to the initial dose 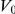 and the qualitative form of the temporal dynamics was maintained as we would expect. Although higher vaccine doses elicited stronger immune responses, there was an upper bound due to saturation of dendritic cells (i.e. the assumption that all local dendritic cells present are stimulated and addition of more vaccine does not lead to further immunological stimulation) but only at a much higher vaccine dose than observed experimentally.

**Figure 4:**
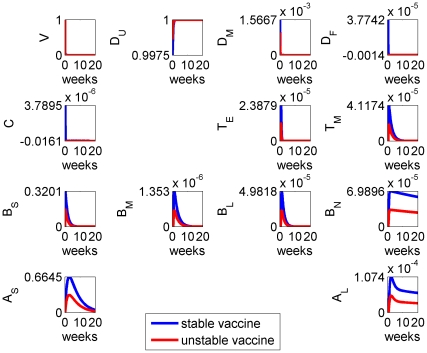
Model results for the system with stable (blue) and unstable (red) vaccine, the latter having a decay rate 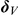 roughly twice that of the former (see Table 2). The benefits of stability are not fully realised until a booster dose is applied (see Figure 5).

We next considered the effect of vaccine stability on the immune response by varying the vaccine decay rate parameter 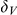: results are given in Figure 4. Vaccine stability had an impact on the magnitude of the immune response, with the more stable antigens stimulating stronger responses in all cell types. However, stability had negligible impact on the overall timing of the immune response.

These model results suggested that antigen dose can compensate for stability within the estimated parameter ranges: specifically, increasing the vaccine dose elicits an immune response comparable with that of a more stable vaccine.

### Effect of vaccine dose and stability on booster vaccination

**Figure 5:**
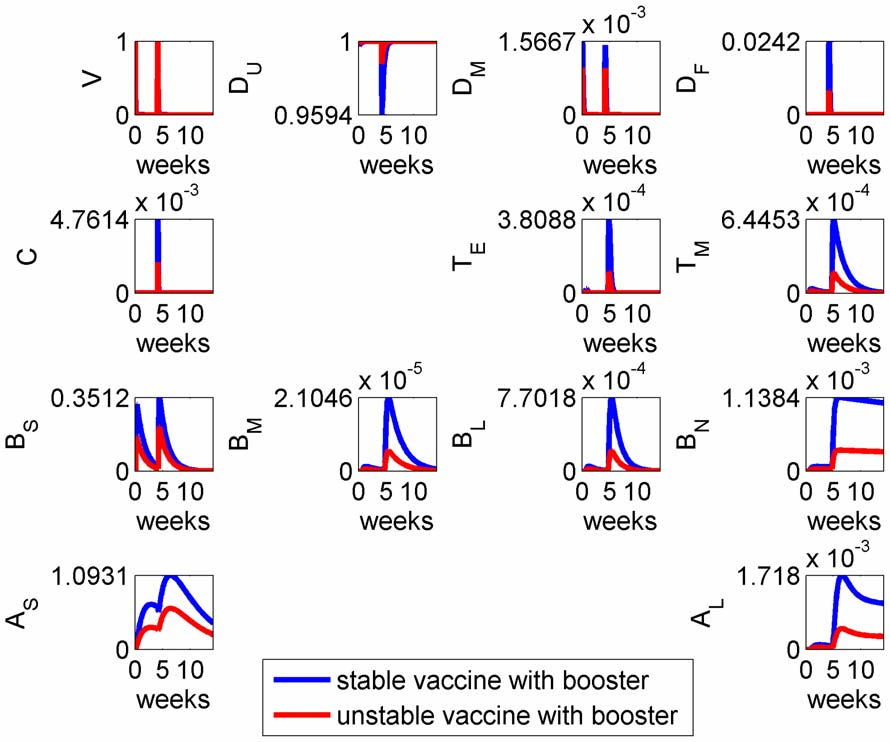
Model results for the system with stable (blue) and unstable (red) vaccine, in response to a second (booster) vaccine dose administered approximately 4 weeks after the initial one.

**Figure 6:**
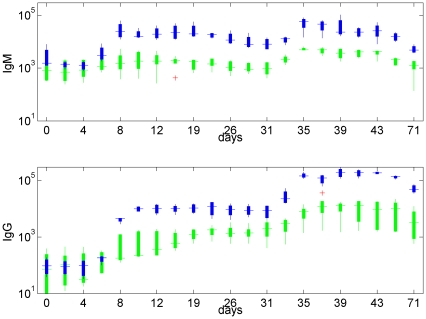
*In-vivo* experimental results for cattle inoculated with a regular dose of vaccine at 0 and 29 days, giving the resultant IgM (left) and IgG (right) levels recorded: (top: blue) normal vaccine producing a regular immune response; (bottom: green) vaccine stimulating the T-cell independent response only. Plots give the median value (central bar), 25th–75th percentile (box) and extreme values (whiskers) unless considered outliers, in which case they are plotted separately (cross) for four (bottom: T-cell independent) or five (top: T-cell dependent) replicates (individual cattle). Data from [9]. Note the significant differences in magnitude between the T-cell dependent and T-cell independent cases. Results presented on a log-scale.

In the field, initial FMD vaccination often elicits transient host protection and repeat vaccination is necessary to maintain protective immunity: this is done at least twice a year where practical (e.g. [15]). Using the model it was possible to investigate the effect and observed cumulative benefit of repeat vaccination with vaccine formulations of differing structural stability. Figure 5 shows the simulation results when a second (identical) vaccine dose 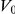 was introduced 29 days after the first, and observed for a further 42 days (71 days in total), reflecting the experimental protocol referred to in Figure 6. We performed this calculation both for stable and unstable vaccine antigens.

The system produced the response in IgM and IgG that would be expected empirically, namely, only a small difference in IgM (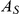) between the first and second dose but a much larger booster effect in IgG (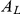) for stable vaccine (blue data in Figure 6). This effect was much larger than can be explained by the sum total of doses alone. It was evident that the model is able to qualitatively capture immunological memory, which is a central feature of the adaptive immune response. As before, stable vaccines elicited a stronger immune response, especially following secondary vaccination. Antibody levels were maintained at a higher level, and for longer, with increasing vaccine stability.

### T-cell dependent responses – comparison with experimental results

Figure 6 shows experimental IgM and IgG levels in cattle immunised twice – an initial dose at 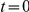 and an equal booster dose at 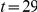 days – with two different vaccines. One vaccine was modelled on a conventional, commercial vaccine (data plotted in blue) that initiates the full immune response. The second was a modified vaccine (data plotted in green) that is designed not to fully initiate a T-cell dependent response. We note that in the latter case there was still some generation of IgG-secreting B-cells, and thus IgG (Figure 6, bottom right), although much reduced. The principle difference is a significant reduction in the IgG response to a booster dose of vaccine in the absence of a T-cell mediated response.

**Figure 7:**
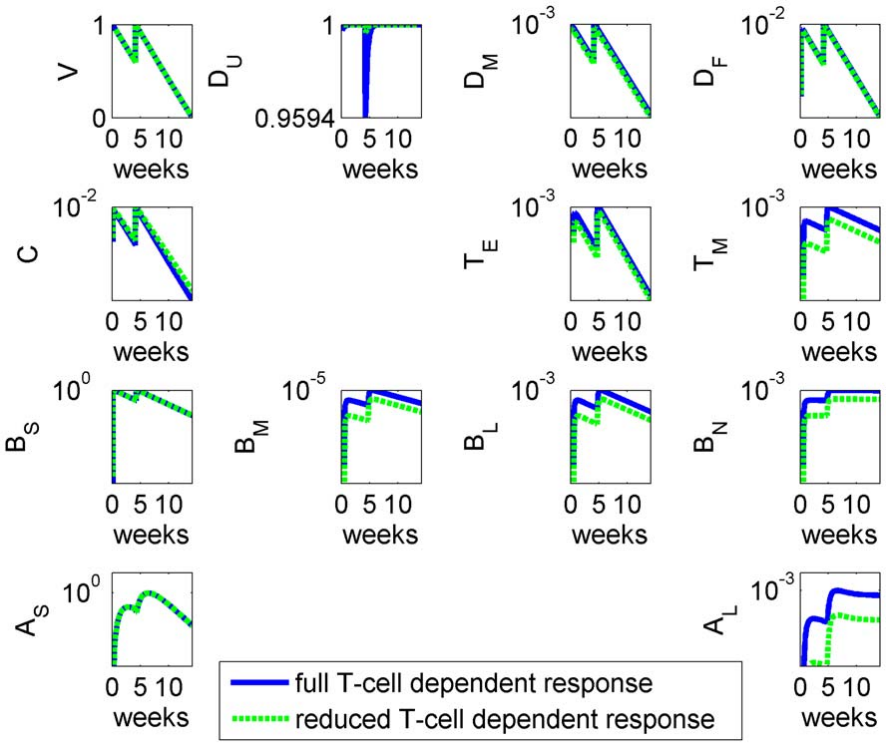
Model results for the full system (blue) and for the system with a reduced T-cell dependent response only (green), where 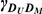 and 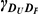 have been reduced to 1% of their original values. All figures present variables on a log scale as percentages of their peak value.

Although the data is best considered qualitatively, using the model we investigated the effect of repeat vaccination with such modified antigens: an inhibited T-cell independent response was represented by reducing 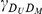 and 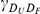 in equations (10)–(13); results are given in Figure 7. Since the T-cell mediated response is an intrinsic component of the long-term (adaptive) pathway that generates IgG, and assumed to require more sustained stimulation, while IgM is produced by the short term pathway only, we considered the data presented in Figure 6 as a guide to the response expected from stable and unstable (i.e. slowly and rapidly decaying) vaccine.

**Figure 8:**
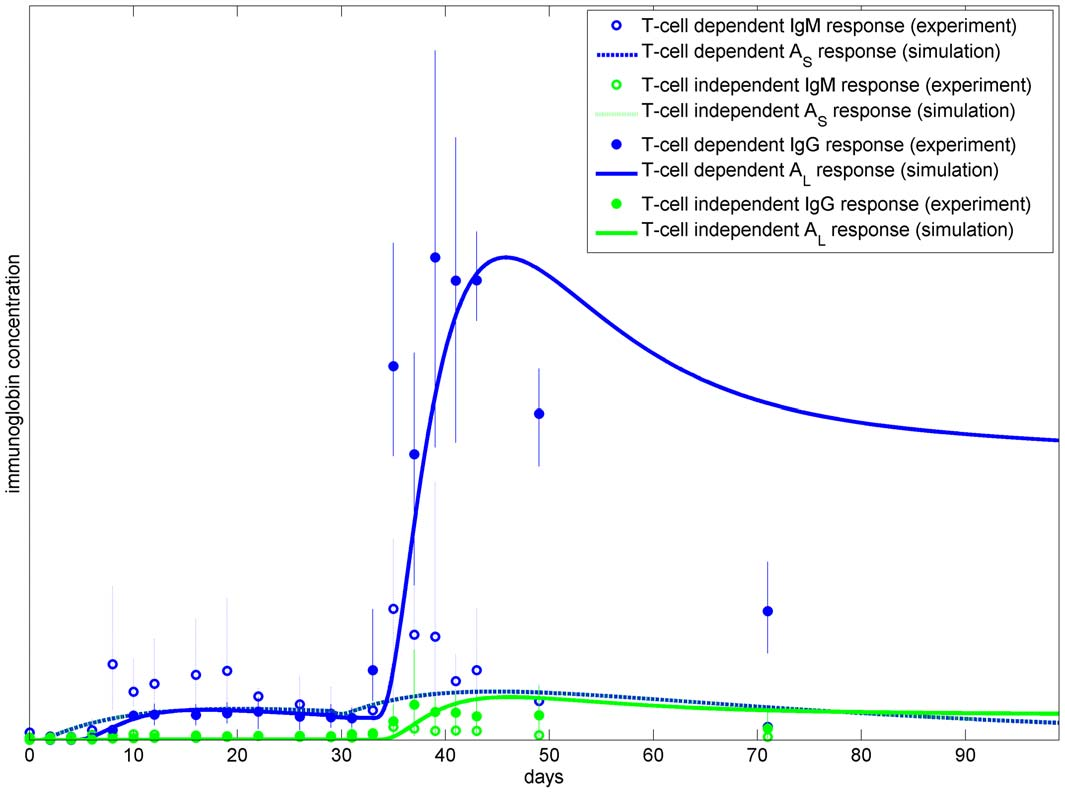
Experimental and simulation results for IgM (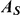) and IgG (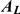) for the full system (blue) and for the system with a reduced T-cell dependent response only (green). Here the mean and range of each of the datasets from Figure 6 are plotted, together with the simulation results from Figure 7, with the model outputs suitably scaled (the peak of the experimental mean for each of the two immunoglobulins matched by the peak of the full T-dependent system).

Although the model only aims to qualitatively replicate observed dynamics, we have plotted experimental results and simulation results together in Figure 8 for comparison. Bearing in mind that the success of the experimental protocol in removing T-cell dependence is unknown, we believe that the model is also able to produce good agreement with the observations for this scenario.

### Sensitivity analysis

We have shown that the model for the within-host immune responses to immunisation with FMDV vaccine antigens of differing stability can lead to realistic qualitative behaviours using plausible sets of estimated model parameters. This indicates that the mathematical model is capable of successfully reflecting the inherent dynamics of the immunological response. However, our model was complex, involving a large number of state variables (13) and model parameters (34). The notional values for the model parameters were estimates but, inevitably, there was some range of uncertainty within which their precise values actually lie. When structural complexity of a mathematical model is allied with model parameter uncertainty it is useful and desirable to be able to systematically assess the likely variation in the results of the model in order to establish whether the sorts of behaviours seen in the output are robust over a broad range of parameter choices. For models to be useful the successful application of such an uncertainty assessment gives confidence that the observed output is not simply a fortuitous combination of model parameters.

**Figure 9:**
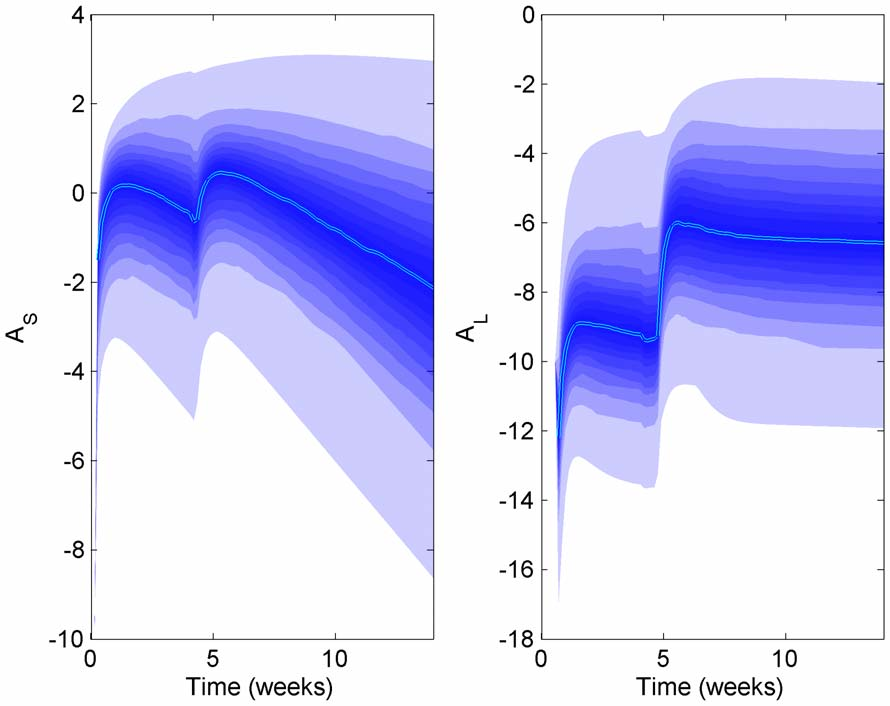
LHS applied to the immunological model with parameter ranging from 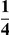 to 4 times the nominal values shows that qualitative behaviour is maintained. Here the median (solid line) is plotted together with the range of possible results, in 5 percentile steps (shaded) from 410 replicates (axes upper bound set at maximum of 95^th^ percentile range) on a log scale.

Figure 9 shows the sensitivity analysis for a LHS scaling factor of 4 (i.e. each parameter is uniformly distributed between 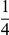 to 4 times its estimated value). It is evident that the qualitative behaviour of the model was preserved for up to this multiple; comparable IgM (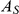) responses following initial and booster vaccination were seen and a significant enhancement in the magnitude of IgG (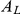) response was maintained. Other calculations (not shown) indicated consistent model behaviour up to multiples of at least 6. This indicates that the model of the T-cell independent and T-cell dependent antibody responses is robust against large uncertainty in the model parameters.

**Figure 10:**
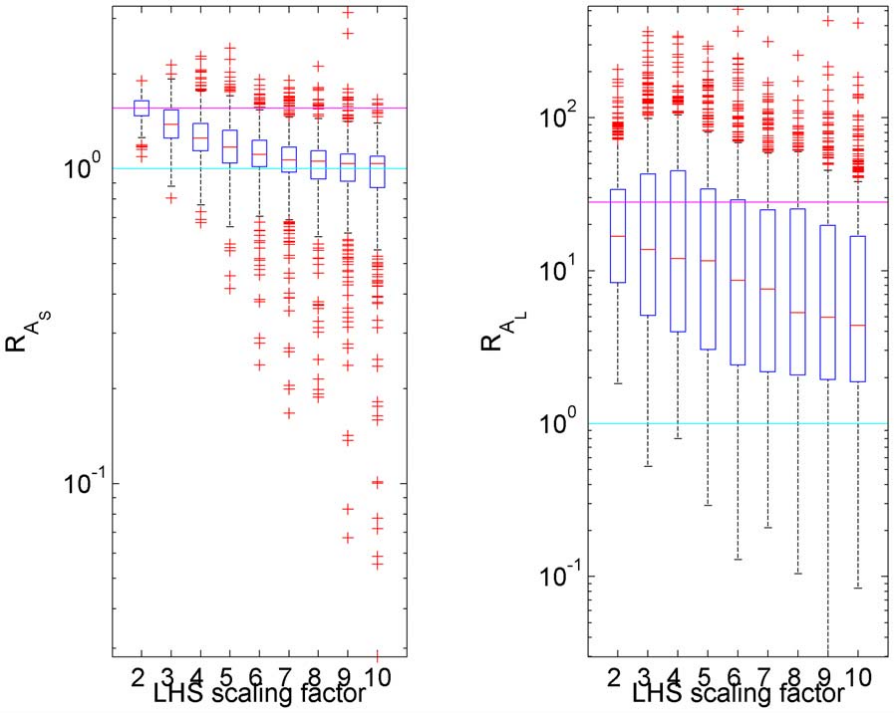
The peaks ratio for IgM and IgG of replicates with variable parameter values selected using LHS, for different multiples. Plots give the median (red bar), 25–75th percentile (box plot), non-outlier range (whiskers) and outliers (red cross) for each multiple set. In addition the ratio from the estimated parameter set is marked (magenta) together with the value 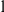 (cyan), where the ratio would switch from an increase to a decrease and which would represent a significant qualitative change in dynamics.

A key feature of the dynamics is the booster effect in IgG (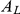), and the metric we used to evaluate whether this is preserved was the ratio of peak responses following the first and second dose of vaccine; explicitly:
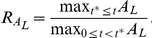
A systematic exploration of the peaks ratio observed for IgM and IgG is shown in Figure 10: ratios greater than 1 were usually observed over parameter multiples up to at least 6 times nominal. As parameter uncertainty increased (i.e. the scaling factor increased) the observed magnitude of theT -cell dependent antibody response decreased, on average (but with higher variability).

## Discussion

We have developed a mathematical model of the bovine immune response to FMDV vaccination, incorporating detailed representations of the T-cell independent and T-cell dependent antibody responses. Such models have helped to further our understanding of the mechanisms involved in adaptive immune responses to host challenge with live pathogens and vaccine antigens [16]. The model reflects the interactions of the principle cell types and antibodies that are thought to be responsible for establishing and maintaining immunity within the host. It is concerned with the CD4+ response and does not include the effects of CTL (cytotoxic T lymphocyte CD8+), since at present there is no evidence that FMDV vaccination stimulates a protective CD8+CTL response. Using the model we have been able to address a number of empirical issues relating to FMDV vaccination. Our motivation has been to provide an explanatory framework within which it is possible to connect hypotheses about detailed mechanisms with experimental data. Moreover, we have attempted to identify many of the relevant parameters that govern the interactions and dynamics of the different cell types.

The model is able to replicate *in-vivo* observations of immunoglobulin dynamics, which lends support to the currently hypothesised immunological response mechanism. The model includes two distinct pathways: short term B-cell production and longer production of target T-cells that drive prolonged antibody responses. Analysis of these produces the behaviour consistent with experimental observations, including the timings of the peak in the long and short term antibody responses. The explicit inclusion of clearly identified parameters which govern specific aspects of the system, such as antibody mediated uptake, allow for the influence of each of these to be explored.

We investigated whether it was possible to mitigate deficiencies in vaccine stability by inoculating with a higher dose of vaccine. This is a question of some significant practical importance. The model results suggest that it is possible to compensate for poor stability with increased dose, but empirically it is found that whilst this is true there is a saturation effect [17]. This is replicated in the model although only at much higher doses than those presented here.

Results suggest that vaccine stability may not have a pronounced impact on the timing of the T cell response, but will affect its magnitude – and hence duration. Future work will look in more detail to how these predictions compare with experimental evidence that vaccine produced from different virus serotypes can differentially stimulate T cell responses. In addition, we were able to use the model to account for experimental results involving conventional and modified vaccine formulations. Results presented here motivate experimental study of additional serotypes with assumed differential stability and T-cell response inducing properties.

The system achieves good qualitative agreement with empirical observations of the system response to booster vaccine doses, and suggests that stable vaccine benefits more from multiple doses. We undertook a LHS sensitivity analysis which demonstrated the robustness of the immunological model under significant parameter uncertainty. However, it will be necessary to provide better estimates of some of the parameters in the model: in particular, we are considering how the deficit in data regarding the *local* saturation of DCs can be remedied. We acknowledge that while it would be ideal for all parameters to be derived from experiments using FMDV vaccine in cattle, a number (e.g. those relating to cell production rates) come from other systems. These are the best available at present and, we believe, in each case an appropriate proxy that does not significantly change subsequent results.

The model provides a consistent representation of immune responses to vaccination and will be used to inform future experimental investigations aimed at enhancing commercial vaccine efficacy. Our model can also be utilised to interrogate the immune response to T-dependent (T-D) and T-independent (T-I) antigens. Antigens that require T-cell help to orchestrate a high affinity class-switched serological response are termed T-D antigens. T-I antigens are able to initiate a serological response in the absence of T-cell help but show little germinal centre formation or B-cell memory. There are two types, type I (polyclonal B-cell stimulant) or type II (non-polyclonal stimulant). It has been demonstrated that T-I antigens can be altered, via conjugation of a protein carrier, to produce a T-D immunological response resulting in induction of a more sustained immunological memory response [18], [19]. Our model has the potential to predict the experimental outcome of enhancing T cell stimulation to drive enhanced B cell responses and long term protective antibody responses.

Mathematical modelling of within-host immunological responses to infection with replicating viruses has been valuable in clarifying and describing the most significant immunological interactions and mechanisms [16], [20], [21]. By contrast, equivalent studies that model the interaction of vaccine formulations with host immune systems are much less well developed. Here we have initiated a similar approach to understanding the interaction of vaccine candidates with immunological responses, and we seek to promote the use of mathematical modelling as an integral component of vaccine development.

## Materials and Methods

### Immune system and antigenic stability

We aim to describe the classical adaptive immune response in as simple a way as possible while including all essential components. The system consists of two principle pathways, referred to as the “short-term” and “long-term” pathways, which are described in detail below. The process is driven by the presence of antigen (in this case vaccine capsid), which initiates the production of T-cells and specialist B-cells that are capable of producing the appropriate antibodies. These antibodies combine with free capsid (and, more importantly, with the relevant wildtype virus if it is present in future) to form a complex that is then removed from the system.

Vaccine capsids are designed not to interfere or inhibit any of the immune response processes, unlike most pathogens and many other external agents that the immune system is required to deal with. FMDV vaccines appear successful in this regard, and we therefore consider the system as closed.

### Short-term (T-cell independent) pathway

Following inoculation vaccine capsid (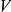) passes, via the lymphatic system, into the lymph nodes. Here the antigen markers on the capsid surface stimulate a cascade of responses that will, in time (following a delay 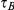), generate targeted B-cells (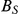) which, in turn, release into the body specifically-targeted antibodies, namely immunoglobin IgM (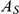) [22]. This antibody binds with the free capsids to form a capsid-antibody complex (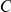) that, although inert, is involved in the long-term pathway (see below). After the antigenic stimulus is removed, the specific B-cell population, and consequently antibody production, will decline to negligible levels over a period of a few weeks. We assume that this pathway has no inherent memory so that any future insults, either by virus or vaccine, will initiate the process anew. The T-cell independent pathway does not produce long lived memory cells [23].

### Long-term (T-cell dependent) pathway

Vaccine capsid is also delivered to the lymph nodes by specialist antigen presenting dendritic cells (DCs), which stimulate a different set of responses to that of the short-term pathway. Unactivated DCs (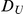) are present throughout the body, and can become activated either by direct uptake of the capsid particles through macropinocytosis (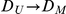) or by uptake of the capsid-antibody complex via the FC receptor mediated process (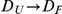). These activated DCs migrate to the lymph-node to present the antigen to naive T-cells, which in time (following a delay 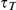), mature to either effector T-cells (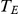) or memory T-cells (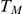). Effector T-cells in turn stimulate development of a new line of differentiated B-cells capable of producing immunoglobin IgG antibodies (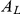); these B-cells may be short-lived (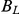) or long-lived plasma (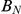) cells, depending on whether they colonize in the bone marrow. The latter are often referred to as “niche” cells since they settle in niches in the bone marrow. In addition, effector T-cells also stimulate memory B-cells (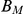) that interact with memory T-cells and vaccine capsid (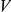) to generate additional effector T-cells: this T-cell dependent antibody response allows for a much more rapid response to “booster” vaccine doses – or exposure to live virus – in future.

The full integrated system, comprising of the short-term and long-term pathways, is illustrated in Figure 1 in terms of the population variables and parameters defined in Tables 1 and 2 respectively, and is in broad agreement with that described by Goodnow *et al.* [24].

### Capsid stability

The structural stability of the complex protein envelope surrounding the RNA of the virus (the capsid envelope), upon which most FMD vaccine formulations are based, appears to be influential in determining the degree of protection afforded by the vaccine. Comparison of the thermal stability of FMD A and SAT2 strains incubated at 49°C indicated an approximately 40-fold difference in the decay rate of live virus [13]. Given the enhanced immunogenicity engendered by more stable capsid particles we use the relative capsid decay rate as a metric of stability in the model.

### Compartmental model

The model equations are derived from the pathways shown in Figure 1 under the assumption of mass action laws and exponential decay where appropriate. The variables represent concentrations i.e. cell number per unit volume. Most parameters represent rates, either production (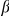), conversion (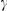) or decay (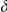). In the absence of data on exactly how delays in the system are distributed, we model the time for B-cell and T-cell generation as simple discrete temporal delays.

The temporal evolution of the vaccine antigen is determined by its decay rate, uptake by IgM (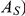, uptake by unactivated DCs (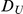) and uptake by IgG (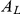), which are explicitly given by:
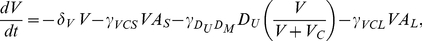
(1)The decay rate parameter 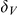 describes the stability of the vaccine capsid (with a half-life given by 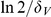). Here 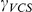 and 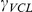 are the rate at which vaccine-IgM complex and vaccine-IgG complex form respectively, while 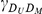 is the rate of vaccine uptake by micropinocytosis.

Although only a tiny proportion of DCs will be activated through vaccination, it is expected that local saturation will occur temporarily. We therefore assume that there exists a preferred inactivated DC concentration to which levels slowly return following uptake, and that the activation rate of DCs obeys Michaelis-Menten kinetics. Unactivated DCs (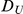) are taken up by antigen (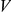) or complex (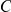) so that:
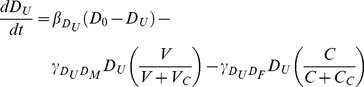
(2)Here 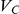 and 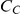 are the level of vaccine and complex respectively resulting in 50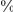 saturation of unactivated DCs. 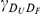 is the rate of uptake of complex by the FC activated pathway, while 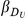 is the rate of dendritic cell migration.

Under the assumption that the level of vaccine in the lymph nodes is proportional to the total level, the dynamics of the short-term antibody secreting B-cells and subsequent short-term IgM concentration are given by
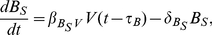
(3)

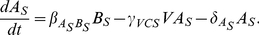
(4)

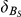
 and 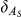 are the natural decay/removal rate of short term antibody secreting cells (ASCs) and IgM respectively, while 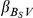 and 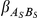 are the rate of production of short-term ASC induced by vaccine and the rate of IgM production by short-term ASC. The production of these B-cells takes a time 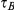 and is thus dependent on the vaccine concentration at a time in the past. IgM is produced in proportion to the number of B-cells and is lost through binding with vaccine antigen and through decay. In the absence of vaccine 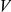, both 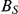 and 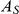 decay to zero as expected.

The generation of the vaccine–antibody complex through the long-term pathway is a result of vaccine binding with IgM and IgG, with loss as a result of uptake by unactivated DCs and natural decay. We do not distinguish between vaccine–IgM and vaccine–IgG complex, since once they are formed the properties of these complexes are very similar. DCs are activated by vaccine uptake via macropinocytosis or via FC activation, but with saturation, and lost through decay, giving:
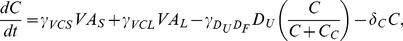
(5)

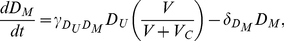
(6)

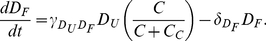
(7)Here 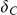, 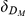 and 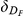 are the decay rates of complex, macropinacytosis-activated DCs and FC-activated DCs respectively.

The presentation of vaccine or complex derived antigenic peptides by activated DCs to naive T-cells generates two classes of T-cells, with the differentiation of naive T-cells assumed to take a time 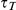. Furthermore, memory T-cells can be converted to effector T-cells by memory B-cells in the presence of vaccine, while memory B-cells themselves are produced in the presence of effector T cells. B cell development is supported by T helper cells.

The rate of decay of effector T-cells, memory T-cells and memory B-cells is given by 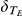, 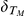 and 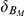 respectively, so that:
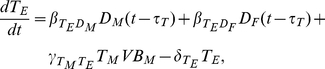
(8)

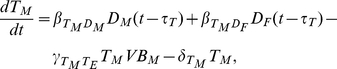
(9)and
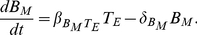
(10)Here 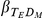 and 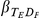 are the rate of effector T-cell production by macropinocytosis activated DCs and FC activated DCs respectively, 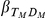 and 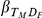 are the rate of memory T-cell production by macropinocytosis activated DCs and FC activated DCs respectively, 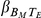 is the rate of memory B-cell production and 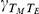 is the rate at which memory T-cells are converted to effector T-cells.

Effector T-cells give rise to B-cells, a proportion of which settle in the bone marrow where their life span is significantly enhanced (long-lived plasma cells). IgG is produced by both types of T-cell dependent B-cells and can form complex with vaccine, giving:
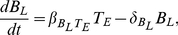
(11)

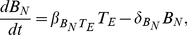
(12)

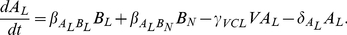
(13)It is assumed that negligible IgG is produced by the short-term pathway, so that 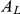 represent only T-cell dependent immunoglobulin. The rates 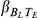, 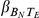, 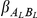 and 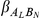 represent short term T-cell dependent ASC production, long-term T-cell dependent ASC production, IgG production rate by short-term T-cell dependent ASC and IgG production rate by long-term T-cell dependent ASC respectively; 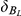, 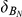 and 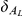 are the rates at which short-term T-cell dependent ASC, long-term T-cell dependent ASC and IgG decay.

Vaccine antigen (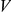) is first introduced as an impulse at time 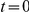 into a system of DCs (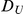), and we therefore impose the following initial conditions:
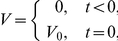
and 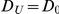 for 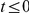, with all other variables equal to zero. The system is defined completely by the closed equations (1)–(13) and these initial conditions. When a “booster” vaccine dose is applied at time 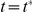, we introduce an impulse of the form
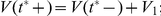
this means that immediately after the timepoint 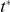 the value of 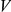 has increased by a quantity 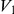 compared to immediately before time 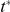. All model variables are given in Tables 1.

### Parameter estimates

The immunology of FMDV has been intensively studied in a variety of *in-vivo* and *in-vitro* contexts. Because of this, and perhaps unusually for an immunological system, we are able to make good estimates for many of the parameters in our detailed model.

Parameter values for the immune model are taken from the literature or estimated from recent experimental results at the Institute for Animal Health – Pirbright Laboratory; where possible, we have sought to do so from experiments relating to FMDV infection in cattle or inoculation with antigens. The nominal parameters for our model are summarised in Table 2, together with sources for each parameter estimate. Although estimates for a number parameters are not available – and there is no way at present to extract these numbers experimentally – it is still possible to make progress by numerical simulation and analysis of the model without them. We have undertaken sensitivity analysis to capture the uncertainty these introduce, and noted which parameters primarily result in the scaling of certain variables. In what follows we therefore restrict our attention to the relative rather than absolute levels of IgG (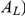 and IgM (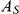) in response to different scenarios, and when comparing output to experimental data.

### Sensitivity analysis

A systematic procedure for investigating the range of behaviours in complex dynamic models is Latin Hypercube Sampling (LHS) – see [25]. It is a statistical analysis technique that permits a comprehensive exploration of the model parameter space in a computationally efficient manner. Its efficiency stems from the fact that each value of each parameter is used only once. The model is run for different combinations of model parameters and the outputs can be described by basic descriptive statistics, though here we are primarily focussed on the robustness of the qualitative behaviours of the model. Essentially the issue is one of investigating the effect of uncertainty in determining model parameters on the model outputs. It is not our intention to review the LHS methodology here, rather we note that it has been successfully applied to deterministic mathematical epidemiology models of comparable size and complexity to the immunological model described above [26], [27] and in systems engineering applications as well [25].

The application of LHS to our immunological model was straightforward and we assumed that the model parameters have uniformly distributed probability distribution functions (pdfs) spanning a range from a given minimum to a given maximum; at this stage we have no other information that permits more complex pdfs to be proposed so we used the simplest representation. Following the standard LHS procedure we generated an appropriate LHS table, selected the resulting parameter combinations and performed ten times the number of simulations required to fully sample the parameter space [25]. The parameters used in the model are itemised in Table 2.
